# A periosteum-derived cell line to study the role of BMP/TGFβ signaling in periosteal cell behavior and function

**DOI:** 10.3389/fphys.2023.1221152

**Published:** 2023-09-20

**Authors:** Emily R. Moore, David E. Maridas, Laura Gamer, Gavin Chen, Kathryn Burton, Vicki Rosen

**Affiliations:** Department of Developmental Biology, Harvard School of Dental Medicine, Boston, MA, United States

**Keywords:** periosteum, cell line, BMP signaling, TGFβ signaling, mechanotransduction

## Abstract

The periosteum is a thin tissue surrounding each skeletal element that contains stem and progenitor cells involved in bone development, postnatal appositional bone growth, load-induced bone formation, and fracture repair. BMP and TGFβ signaling are important for periosteal activity and periosteal cell behavior, but thorough examination of the influence of these pathways on specific cell populations resident in the periosteum is lacking due to limitations associated with primary periosteal cell isolations and *in vitro* experiments. Here we describe the generation of a novel periosteum-derived clonal cell (PDC) line from postnatal day 14 mice and use it to examine periosteal cell behavior *in vitro*. PDCs exhibit key characteristics of periosteal cells observed during skeletal development, maintenance, and bone repair. Specifically, PDCs express established periosteal markers, can be expanded in culture, demonstrate the ability to differentiate into chondrocytes, osteoblasts, and adipocytes, and exhibit an osteogenic response to physical stimulation. PDCs also engage in BMP and/or TGFβ signaling when treated with the activating ligands BMP2 and TGFβ-1, and in response to mechanical stimulation via fluid shear. We believe that this PDC line will be useful for large-scale, long-term experiments that were not feasible when using primary periosteal cells. Anticipated future uses include advancing our understanding of the signaling interactions that occur during appositional bone growth and fracture repair and developing drug screening platforms to discover novel growth and fracture healing factors.

## Introduction

The periosteum is a thin tissue surrounding each skeletal element that contains stem and progenitor cells involved in bone development, postnatal appositional bone growth, load-induced bone formation, and fracture repair (Colnot, 2009; Wang et al., 2017; Moore et al., 2018a; Moore et al., 2018b; Duchamp De Lageneste et al., 2018; Moore et al., 2019; Ortinau et al., 2019; Moore et al., 2020; Xiao et al., 2020; Ono, 2022). In each of these contexts, bone morphogenetic protein (BMP) and/or transforming growth factor-β (TGFβ) signaling is required for periosteal cell function (Tsuji et al., 2006; Chen et al., 2012; Salazar et al., 2016; Wu et al., 2016; Salazar et al., 2019; Xia et al., 2020). Deficits in periosteal cell presence and activity are linked to severe skeletal abnormalities, highlighting the periosteum’s importance in skeletal health (Tsuji et al., 2006; Shi et al., 2017; Moore et al., 2018a; Duchamp De Lageneste et al., 2018; Moore et al., 2019; Salazar et al., 2019; Wang et al., 2019; Moore et al., 2020). The periosteum’s superior regenerative potential has sparked efforts to identify stem/progenitor cells that can be targeted to generate bone where needed. This pursuit has been complicated by the surprising heterogeneity of stem/progenitor cell populations present in the periosteum (Matthews et al., 2021). Markers that have been utilized to identify periosteal stem/progenitor cells include *Prx1, αSMA, Gli1, Ctsk,* and *Pdgfrα* (Kawanami et al., 2009; Grcevic et al., 2012; Matthews et al., 2014; Shi et al., 2017; Debnath et al., 2018; Duchamp De Lageneste et al., 2018; Ortinau et al., 2019; Esposito et al., 2020; Brown et al., 2022; Jeffery et al., 2022; Xu et al., 2022). Genetic mouse models have been used to characterize cell populations resident in the periosteum, but a consensus has yet to be reached on the population dynamics of periosteal stem/progenitors.

One meaningful approach to analyzing periosteal stem/progenitor cells has been to isolate primary cells from whole periosteum and perform *in vitro* experiments. From these studies we have come to appreciate the heterogeneity of cells resident in the periosteum, and learned that periosteal stem/progenitor cells can be expanded in culture, are multipotent and mechanoresponsive, and have high regenerative potential when implanted *in vivo* (Moore et al., 2018b; Debnath et al., 2018; Moore et al., 2019; Perrin et al., 2021; Brown et al., 2022). However, there are several limitations when working with primary periosteal cells. First, isolating the periosteum is technically challenging. The periosteum is extremely thin and requires a microscope to visually detect and dissect. It is also intimately connected to muscle and connective tissues such that contamination of other cell types during isolation is essentially unavoidable. Second, the cellular yield from periosteal preparations pales in comparison to that of more traditional skeletal cell isolations, such as calvarial osteoblasts or bone marrow stromal cells. The periosteum becomes thinner and harder to physically separate from surrounding tissues with age, so isolating a substantial, purified population from adult mice is especially challenging. Third, the osteogenic behavior of periosteal stem/progenitor cells decreases significantly with just a few passages and survival of the various periosteal cell populations in culture remains uncharacterized (Brown et al., 2022). Lastly, depending on the chosen protocol and because of the periosteum’s heterogeneity, isolations can vary significantly between sessions. Collectively, these limitations have made it difficult to conduct large-scale and long-term experiments investigating periosteal cells with a great degree of reproducibility.

Here, we present the establishment of a novel periosteum-derived clonal cell line that expresses classic periosteal cell markers, is multipotent *in vitro*, engages in BMP/TGFβ signaling, and is responsive to mechanical stimulation. These characteristics are stable with passaging, indicating this cell line can be utilized for large-scale *in vitro* experiments. We anticipate this cell line will greatly advance our understanding of periosteal cells in bone growth and regeneration, as well as the signaling mechanisms involved in these biological processes.

## Materials and methods

### Animals

*Bmp2* ^*lacZ*^ mice were generated in the Rosen Lab and model characteristics and genotyping are previously described (Salazar et al., 2019). Experiments were performed in compliance with the Guide for the Care and Use of Laboratory Animals and were approved by the Harvard Medical Area Institutional Animal Care and Use Committee (IACUC). Mice were housed and cared for in accordance with IACUC standards in an AAALAC-accredited facility. Mice were euthanized via CO_2_ inhalation and cervical dislocation as secondary confirmation in accordance with IACUC standards.

### Periosteal cell isolation and culture

**FIGURE 1:**
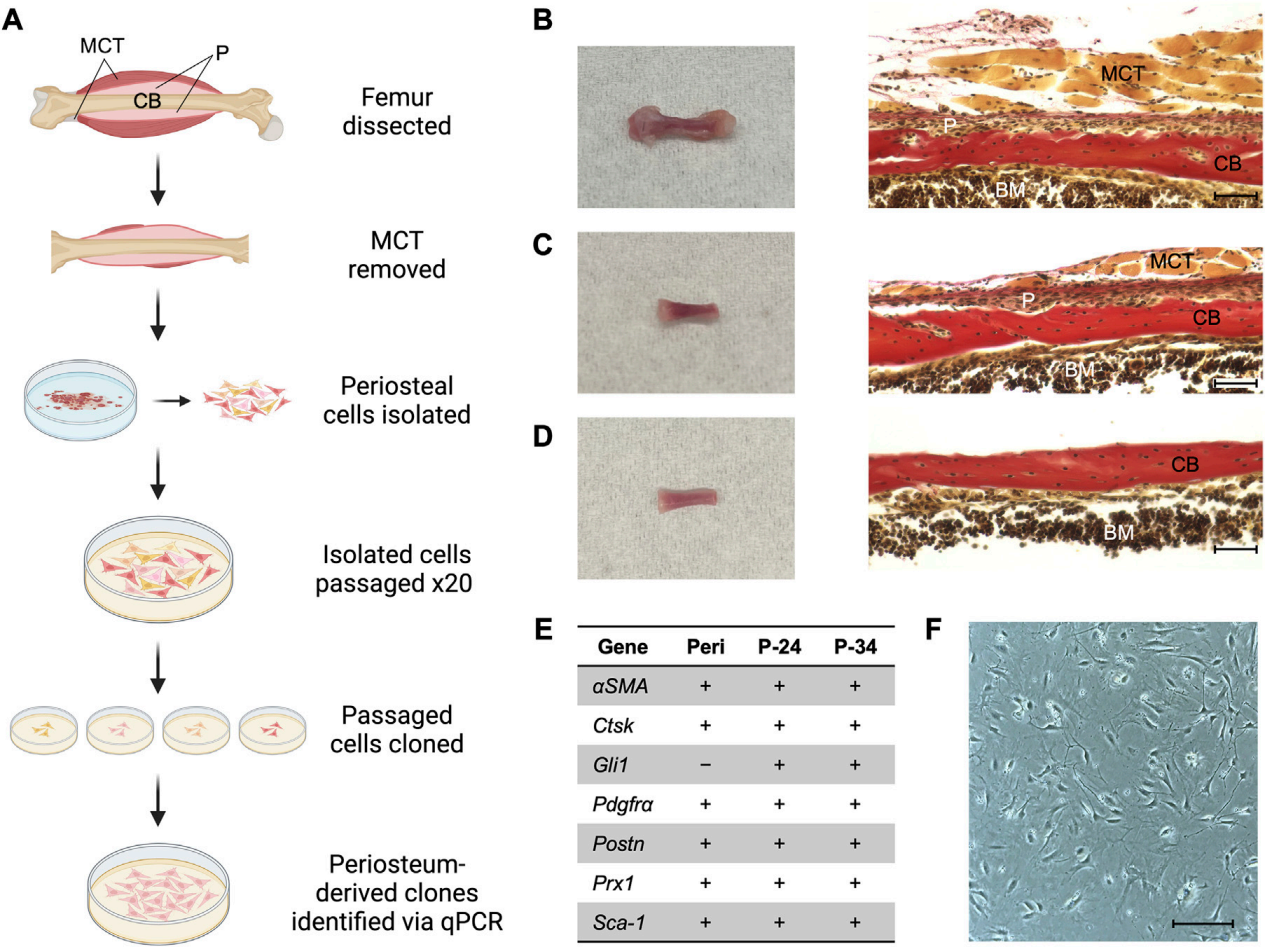
Generating a periosteum-derived cell line. **(A)** Schematic summarizing the steps to generate a line of periosteum-derived cells (PDCs). Created with BioRender.com. Abbreviations: periosteum (P), muscle and connective tissue (MCT), and cortical bone (CB). **(B)** Femur isolated from a P14 *Bmp2* ^*LacZ*^ mouse and corresponding histology stained with Hematoxylin Van Gieson’s to indicate P, MCT, CB, and bone marrow (BM). **(C)** Femur from **(B)** after the epiphyses and most of the MCT were removed under a dissecting microscope. **(D)** Femur from **(C)** after the periosteum was peeled under a dissecting microscope for the cell digest. Images were taken at ×20 magnification and scale bars indicate 100 μm. **(E)** mRNA expression of markers used to identify periosteum-derived clones. Expression was examined in freshly isolated periosteum (Peri) from P14 *Bmp2* ^*LacZ*^ mice and candidate clones at passages 24 (P-24) and 34 (P-34). Denotes markers that are expressed (+) or not detected (−). *n* = 3–4 biological replicates for each group. **(F)** Cell morphology of the clone selected for the PDC line. Image was taken at ×10 magnification and scale bar indicates 100 μm.

Femurs were dissected from two male and two female postnatal day 14 *Bmp2* ^*lacZ*^ mice (see Figures 1A–D). Most of the muscle and connective tissue were removed before placing femurs in PBS on ice. Under a dissecting microscope, a scalpel was used to bisect the growth plates (perpendicular to the longitudinal axis of the femur) to remove most of the epiphyseal ends and associated connective tissue without exposing the bone marrow. Fine-tipped Dumont forceps (Fine Science Tools, 11203-23) were used to separate as much muscle and connective tissue from the femur as possible without disrupting the periosteum. These tissues were gently removed using a scalpel with a #10 blade in a cutting motion parallel to the periosteal surface to avoid pulling off the periosteum. At each epiphyseal end, the scalpel was used to make a single cut around the circumference of the femur 1 mm beneath the growth plate to avoid capturing perichondrium. The scalpel was then used to make a single cut in the periosteum from one epiphyseal end to the other and the periosteum was peeled from the femur using Dumont forceps. The resulting periosteal tissue was incubated in 1 mg/mL Collagenase type I (Millipore, scr103) in sterile PBS in a cell culture incubator (37°C, 5% CO_2_) for 1 h. The digestion solution was then filtered through a 70 μm filter (Corning, 431751) and resulting cells were cultured in MEMα (Gibco, A10490-01) supplemented with 10% fetal bovine serum (FBS) and 1% PenStrep (Gibco, 15140122). Primary cells did not survive if cloned immediately after isolation, so extensive passaging was performed first to eliminate populations incapable of being immortalized. The cells isolated from all four mice were pooled together and passaged twenty times. Cell density never exceeded 80% confluence to avoid potential osteogenic differentiation. Passage 21 (P-21) cells were diluted and seeded at a density of one cell per well in one 96 well plate to establish individual clones. The plate was examined under a microscope to confirm no more than one cell was present in each well. After 2–3 weeks, twelve clones that were viable and proliferated to achieve a confluence greater than 50% were further cultured for two additional passages (P-22, P-23) to observe expansion and viability on a larger scale. Nearly a dozen other wells contained cells, but these populations were excluded based on evidence of cell death and a lack of proliferation. Four of the twelve selected clones proliferated at a comparatively staggering rate and exhibited extensive cell death, so these clones were excluded. The eight remaining candidate clones were reserved in stock vials and examined for mRNA markers of muscle, tendon, and periosteum (Supplementary Figure S1). Stock vials consist of approximately 1 million cells in culture media supplemented with 10% DMSO (Sigma-Aldrich, D2650) and are stored in liquid nitrogen.

### Differentiation culture and staining

Experiments were conducted using both early (P-24–P-26) and later passages (P-32–P-34) to examine consistency. Osteogenic differentiation: media consisted of MEMα supplemented with 20% FBS, 1% PenStrep, 10 mM β-glycerophosphate (Sigma-Aldrich, G9422), and 100 μM Ascorbic acid (Sigma-Aldrich, A4544). Media was changed every 2–3 days and mineral deposition was detected after 3–4 weeks of treatment. Cells were fixed in 10% neutral-buffered formalin (Millipore, HT501128) for 20 min at ambient temperature and incubated in 1.5% Alizarin Red S (Sigma-Aldrich, A5533) solution to detect matrix deposition. Chondrogenic differentiation: periosteal cells were seeded at a density of 100,000 cells per 15 μL to create micromasses. Micromasses were differentiated using the Gibco StemPro Chondrogenesis Differentiation Kit (Gibco, A1007101). Media was changed every 2–3 days and matrix deposition was detected after 1–2 weeks of treatment. Cells were fixed in 4% paraformaldehyde (Electron Microscopy Sciences, 15710) for 15 min at ambient temperature and incubated in 1% Alcian Blue (Electron Microscopy Sciences, 10350) in 0.1 N HCl to detect cartilaginous matrix deposition. Adipogenic differentiation: periosteal cells were treated with DMEM High Glucose (Sigma-Aldrich, D5796) supplemented with 10% FBS, 1% PenStrep, 62.5 mM IBMX (Sigma-Aldrich, I5879), 1 mM Dexamethasone (Sigma-Aldrich, D4902), 20 mM Rosiglitazone (Cayman Chemical, 71742-10), and 2 mM Insulin (Sigma-Aldrich, I6634) for 4 days, followed by treatment with DMEM High Glucose supplemented with 10% FBS, 1% PenStrep, 20 mM Rosiglitazone, and 2 mM Insulin for 3 days. Media was changed every 2 days and lipid formation was detected after 1 week of treatment. Cells were fixed in 10% neutral-buffered formalin for 45 min at ambient temperature and incubated in 0.35% Oil O Red (Sigma-Aldrich, O0625) in isopropanol solution to detect lipid accumulation. Cells were counterstained with 0.1% Crystal Violet (Sigma-Aldrich, C6158). Images were collected at 4X or ×10 magnification using a Keyence BZ-X710 microscope and stitched together using its associated software.

### Ligand treatment

Experiments were conducted using both early (P-24–P-26) and later passages (P-32–P-34) to examine consistency. To detect changes in mRNA transcription: periosteal cells were incubated in MEMα alone for 2 h to synchronize cell activity. Cells were then incubated in MEMα containing 100 ng/mL BMP2 (Genetics Institute) or 1 ng/mL TGFβ-1 (Peprotech, 100-21C) and lysed after 4 h. For Western blotting: periosteal cells were incubated in MEMα alone for 6 h to synchronize cell signaling. Cells were then incubated in MEMα containing 100 ng/mL BMP2 or 1 ng/mL TGFβ-1 and lysed after 45 min of treatment.

### Fluid shear

Experiments were conducted using both early (P-24–P-26) and later passages (P-32–P-34) to examine consistency. Periosteal cells were seeded on 75 × 38 × 1 mm glass slides (Corning, CLS294775X38) and exposed to fluid flow upon exceeding 80% confluence. Fluid shear was applied using a previously described parallel-plate oscillatory fluid flow apparatus (Moore et al., 2018b). Briefly, slides were inserted into chambers containing regular culture media, placed in a cell culture incubator for 30 min to acclimate, and exposed to 1 h of oscillatory fluid flow at 1 Hz with a peak shear stress of 10 dyn/cm^2^. Static controls were similarly placed into chambers and incubated alongside fluid shear samples. Slides were removed from the chambers, rinsed with PBS, and lysed for RTqPCR or Western blotting at the conclusion of flow.

### RTqPCR

RNA was isolated using TRIzol reagent (ThermoFisher, 15596018) and a RNeasy Kit (Qiagen, 74106). RNA was converted to cDNA using the High-Capacity cDNA Reverse Transcription Kit (ThermoFisher, 4368814). qPCR was performed using Faststart universal SYBR Green (Sigma-Aldrich, 4913850001) and a StepOnePlus Real-Time PCR System (Applied Biosciences). mRNA values were normalized to GAPDH or β-actin–housekeeping genes constitutively expressed at high levels–to account for general variability in mRNA expression between samples. Genes that were within 12 cycles of the cycle at which GAPDH reached the threshold for expression were considered expressed in the PDC line. Experimental groups are expressed as a fold change in relation to controls normalized to a value of “1”. Primer sequences are available upon request.

### Western blotting

Protein was isolated using RIPA Buffer (Cell Signaling Technology, 9806S) supplemented with protease and phosphatase inhibitors (ThermoFisher, 78440). Samples were examined by immunoblotting after SDS-PAGE using a 10% Bis-Tris gel (Invitrogen, WG1202BX10). Following transfer, membranes were blocked in TBST containing 5% non-fat dry milk and 5% bovine serum albumin and incubated overnight at 4°C in the following primary antibodies diluted in blocking buffer (1:1000): pSmad1 (Cell Signaling Technology, 13820S), Smad1 (Cell Signaling Technology, 9743S), Smad2/3 (Cell Signaling Technology, 8685S), pSmad2 (Cell Signaling Technology, 3108L), and β-actin (Cell Signaling Technology, 4967S). Membranes were then incubated for 1 h at ambient temperature in an anti-Rabbit IgG HRP-linked secondary antibody (Cell Signaling Technology, 7074S) diluted in blocking buffer (1:2000). Blots were developed using SuperSignal West Femto Maximum Sensitivity Substrate (ThermoFisher, 34095). Images were acquired with a PXi4 Chemiluminescent and Fluorescent Imaging System (Syngene, Bangalore, India) and quantification was performed using ImageJ software (National Institutes of Health).

### Histology

Dissected femurs were fixed in 10% formalin overnight, decalcified in 0.5 M EDTA (VWR, 75800-470) for 1 week, dehydrated, embedded in paraffin, and sectioned in 5–10 μm slices. Slides were rehydrated and stained using Weigert’s Iron Hematoxylin A (VWR, 26044-05), Weigert’s Iron Hematoxylin B (VWR, 26044-15) and Van Gieson’s Solution (VWR, cat #26046-05). Images were collected at ×20 magnification using a Keyence BZ-X710 microscope and its associated software.

### Mycoplasma testing

The clonal cell lines used for this experiment were determined to be negative for *mycoplasma* using the MycoAlert™ *Mycoplasma* Detection Kit (Lonza, LT07-118). Clones were tested the first passage after thawing a stock vial (passage 24, P-24), at P-28 or P-29 during experimentation, and at the conclusion of experiments at P-34.

### Karyotyping

PDCs at passage 28 (P-28) and P-33 were seeded at two different densities on 25 cm^2^ flasks and shipped at ambient temperature overnight to KaryoLogic, Inc. in Durham, NC for karyotyping. Cytogenetic analysis was performed on twenty G-banded metaphase spreads and we consulted with a senior analyst to interpret the findings.

### Statistics

Researchers were blinded to all data analysis. Differences between control and experimental groups were determined using a two-tailed Student’s *t*-test. Values are reported as mean ± SEM, with *p* < 0.05 considered statistically significant. The sample size was selected to achieve a power of at least 80%. Statistical analysis was conducted using GraphPad Prism (San Diego, CA).

## Results

### Generating a periosteum-derived clonal cell (PDC) line

Primary periosteal cells were isolated from postnatal day 14 (P14) *Bmp2* ^*LacZ*^ mice to maximize cell yield and to utilize X-gal staining to detect BMP2. Briefly, periosteal tissue from femurs was pooled and digested to extract primary cells (Figure 1). These cells were passaged 20 times to select for cells that exhibited high-passage potential and then seeded at a density of 1 cell per well to generate clones. Clones that survived and proliferated were examined for mRNA expression of published markers for periosteal (*αSMA*, *Gli1*, *Pdgfrα*, *Ctsk*, *Prx1*, *Pstn*, and *Sca-1*), skeletal muscle (*Mef2c*, *Myh2*, *Myod1*, and *Myog*), and connective tissue (*Scx*, *Tnmd*) cells (Colnot et al., 2012; Grcevic et al., 2012; Debnath et al., 2018; Duchamp De Lageneste et al., 2018; Jo et al., 2019; Matthews et al., 2021; Piasecka et al., 2021). We first examined these markers in freshly isolated periosteum from P14 *Bmp2* ^*LacZ*^ mice. All periosteal markers except *Gli1* were detected and the muscle and connective tissue markers were not detected. Two candidate clones expressed all periosteal markers, and muscle and connective tissue markers were not detected (Supplementary Figure S1). In fact, none of the candidate clones examined expressed *Scx* or *Tnmd*. This mRNA expression profile remained consistent for 10 passages, suggesting the clones are stable with further passaging (Figure 1E). The following experiments were conducted using cells within the 10 passages examined for mRNA expression and findings at different passages were consistent. For simplicity, we highlight data for one clone to describe a periosteum-derived cell (PDC) line in this manuscript, but results were consistent for both clones (Supplementary Figure S2; Figure 1F).

### The PDC line is multipotent *in vitro*

**FIGURE 2:**
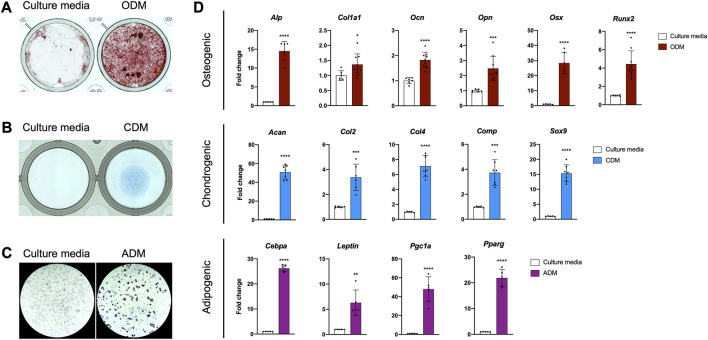
The PDC line is multipotent. **(A)** Alizarin Red staining of PDCs incubated in culture media and osteogenic differentiation media (ODM). **(B)** Alcian Blue staining of PDCs incubated in culture media and chondrogenic differentiation media (CDM). **(C)** Oil Red O staining of PDCs incubated in culture media and adipogenic differentiation media (ADM). Images were collected at ×4 magnification and *n* = 3–4 technical replicates in 3 biological replicates for each group in **(A**–**C)**. **(D)** mRNA expression of genes associated with differentiation. Values are represented as fold changes with DM treatment compared to regular culture media controls. Osteogenic and Chondrogenic values are normalized to *Gapdh* expression, Adipogenic values are normalized to *β-actin* expression. *p < 0.05, **p < 0.01, ***p < 0.001, ****p < 0.0001, n = 6-8 technical replicates in 3 biological replicates for each group. Replicates are represented as individual dots on bar graphs.

Periosteal stem and progenitor cell populations are known to differentiate into osteoblasts, chondrocytes, and adipocytes *in vitro*, so we examined the lineage potential of our line (De Bari et al., 2006; Arnsdorf et al., 2009a; Debnath et al., 2018; Perrin et al., 2021). PDCs incubated in osteogenic differentiation media deposited mineral in 3–4 weeks (Figure 2A). We detected cartilage matrix deposition in PDC micromasses incubated in chondrogenic differentiation media for 1–2 weeks (Figure 2B). Lipid accumulation was observed in PDCs treated with adipogenic differentiation media after 1 week (Figure 2C). mRNA markers for osteogenesis, chondrogenesis, and adipogenesis were significantly upregulated in differentiated PDCs (Figure 2D). These studies indicate the PDC line is multipotent and differentiates into the lineages expected for a periosteal stem cell *in vitro*.

### The PDC line engages in BMP/TGFβ signaling

**FIGURE 3:**
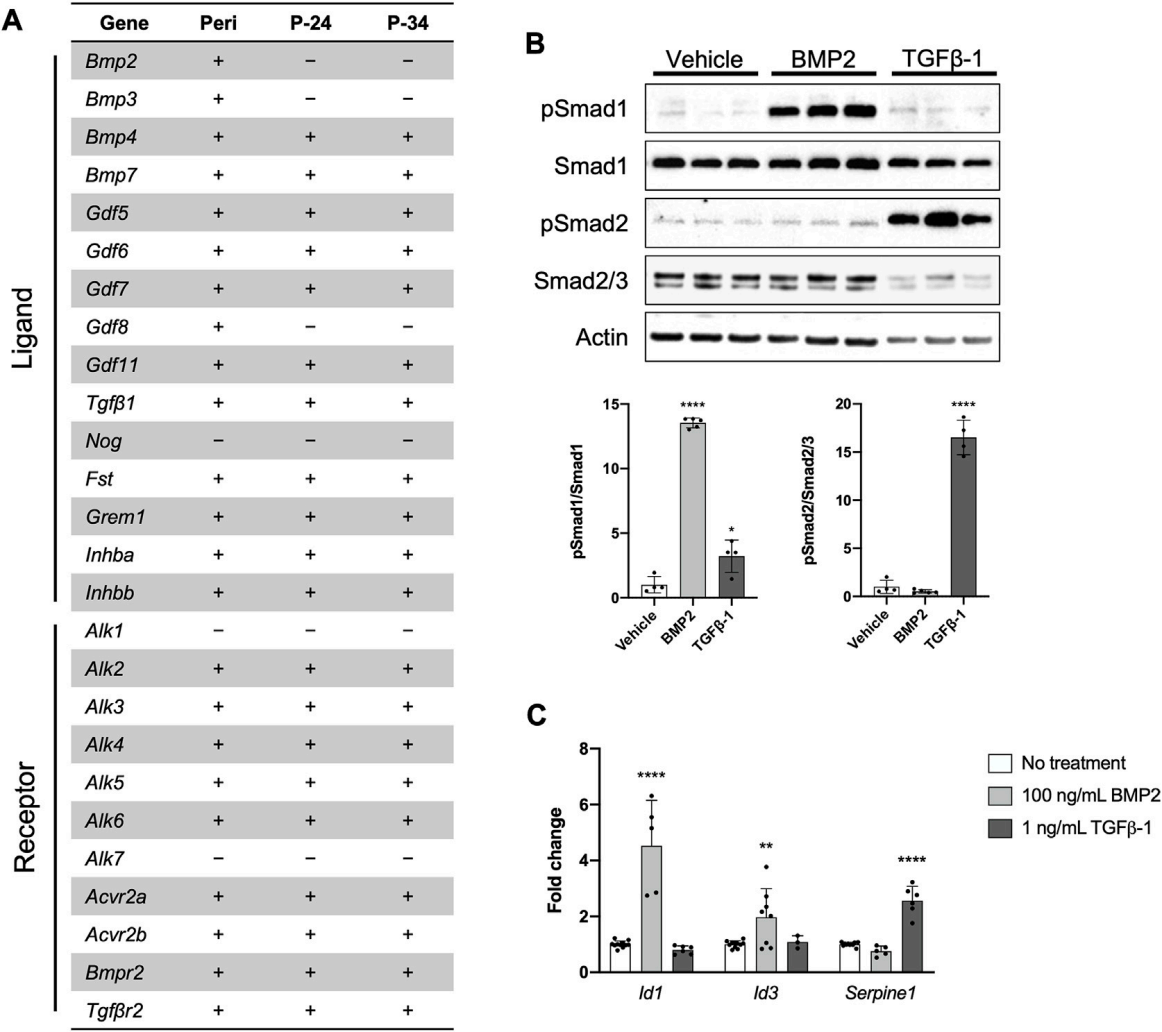
The PDC line engages in BMP/TGFβ signaling. **(A)** Expression of genes associated with the BMP/TGFβ superfamily in freshly isolated periosteum (Peri) from P14 *Bmp2* ^*LacZ*^ mice and PDCs at passage 24 (P-24) and 34 (P-34). Denotes markers that are expressed (+) or not detected (−). *n* = 4–6 technical replicates in 3 biological replicates for each group. **(B)** Representative Western blot image and quantification of changes in total and phosphorylated (p) Smad1 and Smad2/3 with 100 ng/mL BMP2 or 1 ng/mL TGFβ-1 treatment. All values were normalized to Actin expression. *p < 0.05, ****p < 0.0001, n = 4-5 technical replicates in 3 biological replicates for each group. **(C)** Fold changes in mRNA expression of genes associated with activation of BMP and TGFβ signaling in PDCs treated with 100 ng/mL BMP2 or 1 ng/mL TGFβ-1. Changes in expression are represented as fold changes compared to static controls and all values are normalized to *Gapdh* expression. **p < 0.01, ****p < 0.0001, *n* = 3–4 technical replicates in 3 biological replicates for each group. Replicates are represented as individual dots on bar graphs.

Recent work indicates the importance of BMP and/or TGFβ signaling for normal periosteal activity during skeletal development, fracture repair, and load-induced bone formation (Tsuji et al., 2006; Chen et al., 2012; Salazar et al., 2016; Wu et al., 2016; Salazar et al., 2019; Xia et al., 2020). We therefore determined whether our PDC line could be utilized to study these signaling pathways *in vitro*. First, we examined mRNA expression of components in the TGFβ/BMP superfamily in freshly isolated periosteum and early and later passages of the PDC line (Figure 3A). Nearly all the associated components were expressed in the PDC line except for the ligands *Bmp2*, *Bmp3*, *Gdf8*, and *Nog*, and Type I receptors *Alk1* and *Alk7*. This expression profile was consistent between the early and later PDC passages, but we noted some inconsistencies with whole periosteum. Specifically, we detected *Bmp2*, *Bmp3*, and *Gdf8* expression in whole periosteum, which was absent in PDCs. We then examined BMP and TGFβ signaling in the PDC line by treating with ligands known to activate these pathways in periosteal cells. PDCs incubated in media containing recombinant BMP2 exhibited an increased ratio of phosphorylated Smad1 (pSmad1) to total Smad1 compared to vehicle controls, which is indicative of activated canonical BMP signaling (Figure 3B). The ratio of pSmad2/Smad2/3 was unchanged with BMP2 treatment compared to vehicle controls. PDCs incubated in media containing recombinant TGFβ-1 exhibited increased levels of pSmad2/Smad2/3, which is typically associated with activated canonical TGFβ signaling. Interestingly, pSmad1/Smad1 levels were also slightly elevated with TGFβ-1 treatment. When signaling is activated, pSmads bind Smad4 and translocate to the nucleus to trigger transcription of genes associated with canonical BMP (*Id1, Id3*) and TGFβ (*Serpine1*) signaling. We therefore examined changes in mRNA expression of these target genes with treatment (Figure 3C). Indeed, *Id1* and *Id3* were uniquely upregulated with BMP2 treatment and *Serpine1* was upregulated only by TGFβ-1 treatment.

### The PDC line is mechanosensitive

**FIGURE 4:**
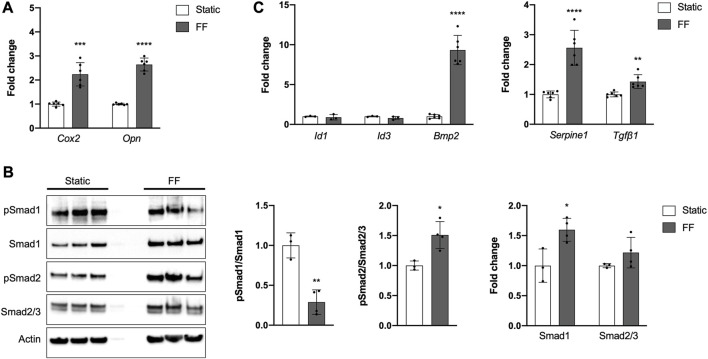
The PDC line is mechanoresponsive. **(A)** Fold change in mRNA expression of genes associated with mechanically-induced osteogenesis under static or fluid flow (FF) conditions. **(B)** Representative Western blot image and quantification of changes in total and phosphorylated (p) Smad1 and Smad2/3 under static or FF conditions. All values are normalized to Actin expression and fold changes are normalized to static controls. *p < 0.05, **p < 0.01, *n* = 3–4 technical replicates in 3 biological replicates for each group. **(C)** Fold change in mRNA expression of genes associated with BMP and TGFβ signaling. Changes in mRNA expression **(A,C)** are represented as fold changes compared to static controls and all values are normalized to *Gapdh* expression. *n* = 4–6 technical replicates in 3 biological replicates for all groups. **p < 0.01, ***p < 0.001, ****p < 0.0001. Replicates are represented as individual dots on bar graphs.

Periosteal cells are known to be mechanosensitive and are important for load-induced bone formation (Arnsdorf et al., 2009a; Moore et al., 2018b; Moore et al., 2019; Xiao et al., 2020). We examined whether our PDC line was mechanosensitive using a custom fluid shear chamber device previously used to study primary periosteal cell mechanotransduction *in vitro* (Moore et al., 2018b). Indeed, PDCs exposed to fluid flow exhibited increases in *Cox2* and *Opn*, genes that are upregulated in bone cells in response to physical stimulation (Figure 4A) (Wadhwa et al., 2002; Ponik and Pavalko, 2004; Arnsdorf et al., 2009b; Hoey et al., 2012; Moore et al., 2018b). TGFβ signaling is believed to be important in periosteal cell mechanotransduction so we examined whether this pathway was upregulated in response to fluid flow (Figures 4B, C) (Raab-Cullen et al., 1994; Klein-Nulend et al., 1995; Nguyen et al., 2020). As expected, pSmad2/Smad2/3 levels increased with fluid flow and mRNA expression of the target genes *Serpine1* and *Tgfβ1* also increased. The role of BMP signaling in load-induced bone formation and periosteal cell mechanotransduction is less clear (Kopf et al., 2012; McBride-Gagyi et al., 2015). In our system, we found that pSmad1/Smad1 levels decreased in PDCs exposed to fluid flow and *Id1* and *Id3* expression was unchanged compared to static controls. However, *Bmp2* expression was significantly elevated with application of. The decreased pSmad1/Smad1 levels seen in PDCs exposed to fluid flow are in part due to a significant increase in total Smad1. Smad2/3 levels were comparable between static and fluid flow groups.

### Chromosomal abnormalities in the PDC line

Standard G-banded karyotyping was performed on PDCs at P-28 and P-33 to determine what chromosomal abnormalities, which are expected for immortalized cell lines, were present (Stepanenko and Dmitrenko, 2015). Abnormal karyotypes were present in both passages, with several consistent observations between the varying spreads. At both passages multiple polysomies, or additional copies of chromosomes, were observed and only one copy of Chromosome 14 was present. P-28 cells exhibited a modal chromosome number of 72, ranging from 63 to 75 across the spreads. P-33 cells had a modal chromosome number of 72 ranging from 68 to 74 across spreads. Markers, or structurally abnormal chromosomes that cannot be unambiguously identified by conventional banding cytogenetics, were also detected. P-28 cells exhibited 2-6 markers among spreads and this range increased to 4-10 in P-33 cells. A single dicentric chromosome was observed in two spreads of P-33 cells. As dicentrics can have difficulty passing through mitosis, it is recommended that the PDC line be used up to P-34, the highest passage we validated. Further validation is encouraged when using cells beyond P-34. X and Y sex chromosomes were present, indicating the PDC line was derived from a male mouse.

## Discussion

In this work we present a new tool to study periosteal cell behavior and signaling *in vitro*. Our periosteum-derived clonal cell line expresses established periosteal markers, engages in signaling pathways known to be important for periosteal cell osteogenesis, and exhibits an osteogenic response to physical stimulation. More importantly, these characteristics are stable with extensive passaging. The features of PDCs address many of the issues associated with using primary periosteal cells for *in vitro* experiments. The purity and yield of primary periosteal cell isolations can vary drastically with animal age, approach, and personnel so a clonal line provides much needed standardization in the periosteum field. By creating a clonal line, we also avoided concerns with contamination of muscle or connective tissue cells. Osteogenic and chondrogenic behavior can become limited with passage in primary cell populations, but we found this behavior was consistent with passaging in the PDC line. Thus, the PDC line can be expanded for large-scale and long-term *in vitro* experiments, such as drug screens, bulk RNA sequencing, and allograft design. We therefore conclude that our PDC line can be utilized *in vitro* to better understand periosteal cell activity and inform *in vivo* studies.

We focused on key features to assess utility of our PDCs, but further experiments are required to fully characterize this line and determine its potential uses. Our PDCs are clonal, viable with extensive passaging, and multipotent, but this only confirms stem cell-like qualities *in vitro*. PDCs express mRNA for markers attributed to periosteal stem/progenitor cells such as *Sca-1*, *Cd29*, *Cd51*, and *Cd105* (Supplementary Figure S1D), but *in vivo* implantation studies and cell-surface marker analysis are necessary to determine whether this is truly a stem cell line (Debnath et al., 2018; Deveza et al., 2018; Duchamp De Lageneste et al., 2018; Bradaschia-Correa et al., 2019; Ortinau et al., 2019; Matthews et al., 2021; Jeffery et al., 2022; Julien et al., 2022; Perrin and Colnot, 2022). It is unknown if our PDCs represent an abundant or rare population found *in vivo*, and it is likely slowly dividing stem/progenitor cells were lost in the process of extensive passaging and cloning. Existing studies have focused on cells selected using a single periosteal marker, but our mRNA expression analysis indicates PDCs express many of these markers (Figure 1E). Considering the heterogenous nature of the periosteum, it is possible that periosteal cells express multiple markers at low levels *in vivo*. Through cloning, we may have captured a population enriched in several periosteal markers. It is equally possible that culturing itself altered transcription. Our mRNA analysis also showed that PDCs express *Gli1*, which was absent in periosteal tissue from which the PDC line was derived. We speculate this is due to enrichment in PDCs compared to whole periosteum. The presence of *Gli1*-expressing cells is known to diminish with age, so it is possible that by P14 there are too few cells for RTqPCR detection (Shi et al., 2017; Shi et al., 2021). One final limitation is that we only examined BMP and TGFβ signaling in PDCs. There are other signaling pathways involved in periosteal cell activation and differentiation that will require initial characterization before determining the full experimental utility of the line. We recognize that our line may not be appropriate for all periosteum studies, especially those that require a heterogenous population or slowly dividing periosteal stem/progenitor populations. However, we can conclude at this time that our PDC line will be useful for examining periosteal cell activation, differentiation, and BMP/TGFβ signaling, events central to appositional growth, fracture repair, and load-induced bone formation.

We examined BMP and TGFβ signaling in PDCs because these pathways are known to be important for periosteal cell activation and differentiation. Treatment with recombinant BMP2 and TGFβ-1 activated signaling and corresponding gene transcription (Figures 3B, C). BMP2 uniquely activated canonical BMP signaling. TGFβ-1 activated canonical TGFβ signaling as expected, but also slightly increased pSmad1/Smad1 levels which are typically associated with BMP signaling. However, this increase did not correspond with upregulated BMP signaling, as we observed no changes in *Id1* and *Id3* transcription. It is possible that PDCs can be activated by other ligands in the BMP/TGFβ pathway: we highlighted BMP2 and TGFβ-1 because their importance in the periosteum is established (Tsuji et al., 2006; Salazar et al., 2019; Nguyen et al., 2020).

Trends in mRNA expression of BMP/TGFβ pathway components in PDCs are consistent with what has been found in other skeletal cells (Salazar et al., 2016; Wu et al., 2016; Lademann et al., 2020). The PDC expression profile is largely consistent with that of whole periosteal tissue from which PDCs were derived, with a few exceptions. *Gdf8*, or Myostatin, is expressed in whole periosteum, but absent from PDCs. We attribute this to muscle contamination when isolating periosteum. In fact, 4 of the 8 candidate clones for the PDC line expressed muscle cell markers, highlighting the risk of muscle cell contamination in periosteal preps. *Bmp2* and *Bmp3* are expressed in whole periosteum but not detected in PDCs. *Bmp3* has been found to be highly expressed in osteoblasts and osteocytes but absent from bone marrow stromal cells and stem and progenitor cells in the periosteum (Kokabu and Rosen, 2018). Osteoblasts and their precursors are present near the periosteum-bone interface, especially during rapid postnatal appositional growth, so it is not surprising to see *Bmp3* expression in whole periosteum isolated at P14. We previously detected *Bmp2*-expressing cells residing in the cambium layer of the periosteum very near the bone surface (Salazar et al., 2019). We speculate these cells are differentiating cells committed to an osteogenic lineage. Thus, the lack of *Bmp2* and *Bmp3* expression combined with our multipotency data (Figure 2) suggests our PDCs are not committed to an osteogenic lineage and exhibit a degree of stemness.

Interestingly, we detected *Bmp2* mRNA expression in PDCs seeded for fluid flow studies (Figure 4C). We initially suspected the shift was due to increased cell density, but we did not detect *Bmp2* in PDCs seeded on tissue culture plates ranging from 50%–100% confluence (data not shown). Another possible explanation is PDCs respond to the increased substrate stiffness when seeded on glass slides. Periosteal cells and other mechanoresponsive bone cells exhibit changes with increased substrate stiffness and osteocyte-like cells must be seeded on collagen-coated glass slides to prevent de-differentiation (Chen and Jacobs, 2013; Wang et al., 2022; Mattei et al., 2015; Zhang et al., 2017; Navarrete et al., 2017; K et al., 2010). *Bmp2* expression increased in PDCs exposed to fluid flow, further suggesting a role for BMP2 in PDC mechanosensation (Figure 4C). We derived the PDC line from heterozygous *Bmp2* ^*LacZ*^ mice so that X-gal staining could be used to visualize BMP2, for which there is no working antibody. In these mice, one copy of *Bmp2* is replaced with a LacZ cassette and mice develop normally with the remaining wildtype allele (Gamer et al., 2018; Salazar et al., 2019). We confirmed the PDCs contain the LacZ gene (Supplementary Figure S6) and X-gal staining can therefore be used to identify PDCs implanted *in vivo* or in co-cultures, as well as to visualize BMP2 secretion by PDCs, for example. Using this visual tool, we intend to further examine the role of periosteal BMP2 in the context of mechanotransduction.

We also evaluated BMP and TGFβ signaling in the context of PDC mechanotransduction. Canonical TGFβ signaling was activated in PDCs in response to fluid flow, which is consistent with existing *in vitro* and *in vivo* work examining mechanoresponsive skeletal cells (Figures 4B, C) (Raab-Cullen et al., 1994; Klein-Nulend et al., 1995; Sakai et al., 1998; Vermeulen et al., 2020; Monteiro et al., 2021). For canonical BMP signaling, pSmad1/Smad1 levels were downregulated and mRNA transcription of downstream targets was unchanged in response to fluid flow (Figures 4B, C). Interestingly, Smad1 levels and *Bmp2* expression increased in PDCs exposed to fluid flow, suggesting the cells are being primed for BMP signaling. Based on these results, we speculate that TGFβ signaling plays a role in mechanotransduction and activation of PDCs, and BMP signaling facilitates subsequent differentiation of PDCs. It is important to note that for fluid flow studies we did not serum-starve PDCs to synchronize the cells and deplete basal signaling like we did for the ligand treatment experiments (Figure 3B). It is possible that standard culture media elevates basal BMP signaling in PDCs such that our current setup cannot capture changes in response to fluid flow. Moreover, BMP signaling is time-sensitive and context-dependent such that protocol optimization is required to confidently detect cellular changes (Greenfeld et al., 2021; Komorowski, 2022). We tested PDCs using a protocol established for primary periosteal cell mechanotransduction studies, but this is not necessarily the optimal timing to observe BMP signaling. In future studies, we will interrogate signaling under different PDC culture conditions and at multiple timepoints to get a more accurate depiction of fluid flow-induced BMP signaling.

In addition to advancing our understanding of periosteal cell behavior and signaling in normal programs of skeletal development, growth, and repair, we anticipate the PDC line will be invaluable to address other questions regarding periosteal activity. We previously generated a genetic mouse model that dampens periosteal BMP signaling, resulting in disrupted appositional growth and a thin bone phenotype in mutants (Salazar et al., 2019). Primary periosteal cells isolated from these mice do not survive in culture, so we intend to utilize the PDC line and CRISPR/Cas9 tools to identify participating periosteal cell populations and mechanisms of osteogenic differentiation in appositional growth. The CRISPR/Cas9 system can further be used in PDCs to examine differences between periosteal stem and progenitor cell populations, as well as inform factors that drive intramembranous *versus* endochondral ossification. These two areas of investigation are critical to understanding the periosteum dynamics that direct unique cellular responses for different biological processes. During fracture repair, the periosteum expands and becomes the predominant source of chondrocytes and osteoblasts that direct reparative bone formation (Colnot, 2009; Duchamp De Lageneste et al., 2018). The PDC line expresses many of the markers thought to be indicative of periosteal stem/progenitor cells involved in repair (Figure 1E) and differentiates into chondrocytes and osteoblasts (Figure 2). Primary periosteal cells have been successfully transplanted in mouse models to study fracture repair *in vivo* (Perrin et al., 2021). We anticipate PDCs will not only successfully transplant *in vivo*, but having a standardized, consistent source of periosteal cells will greatly facilitate the technical aspects of fracture repair and non-union experiments in mouse models. Lastly, we expect the PDC line can be utilized for large-scale experiments like RNAseq and drug screens to provide further insight into periosteal cell behavior to identify targets for anabolic therapeutics that augment bone formation. Considering the wide range of potential uses, we believe this PDC line will significantly advance *in vitro* and *in vivo* investigation of the periosteum.

## Data Availability

The original contributions presented in the study are included in the article/Supplementary Material, further inquiries can be directed to the corresponding author.
