# Supplementary figures and images for: A periosteum-derived cell line to study the role of BMP/TGFβ signaling in periosteal cell behavior and function
Source: Front Physiol. 2023 Sep 20;14:1221152. doi: 10.3389/fphys.2023.1221152 (PMC10547901; doi:10.3389/fphys.2023.1221152)

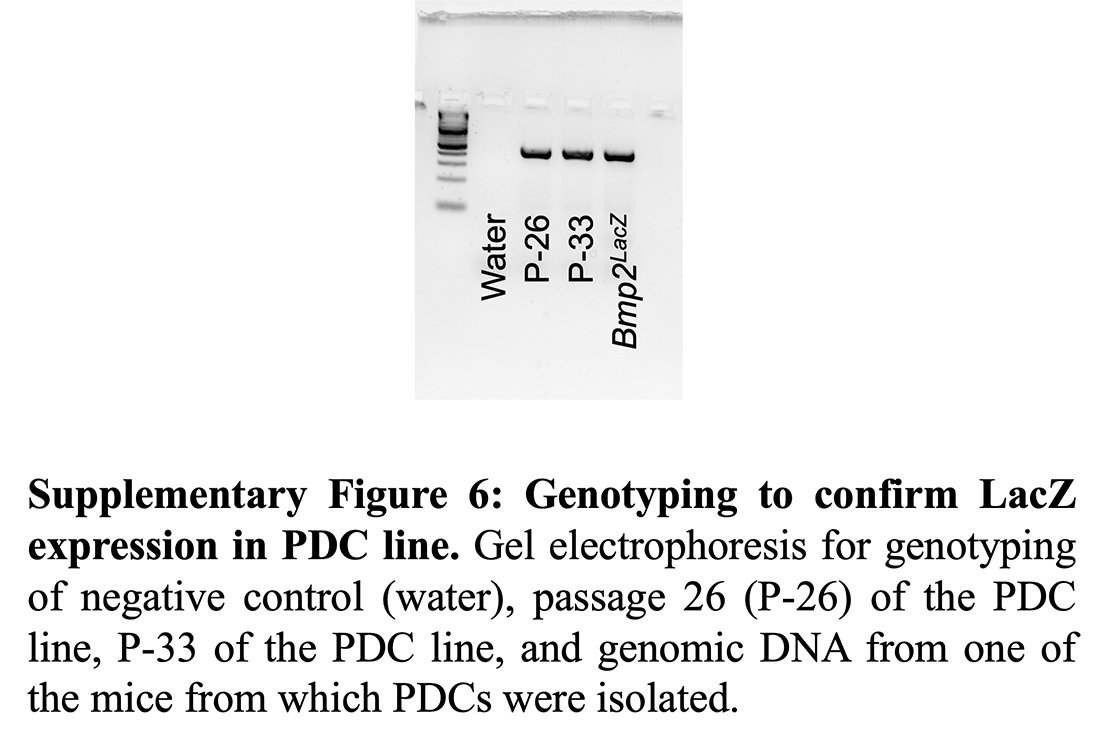

Supplement: Supplementary file 1 [file Image6.TIF]

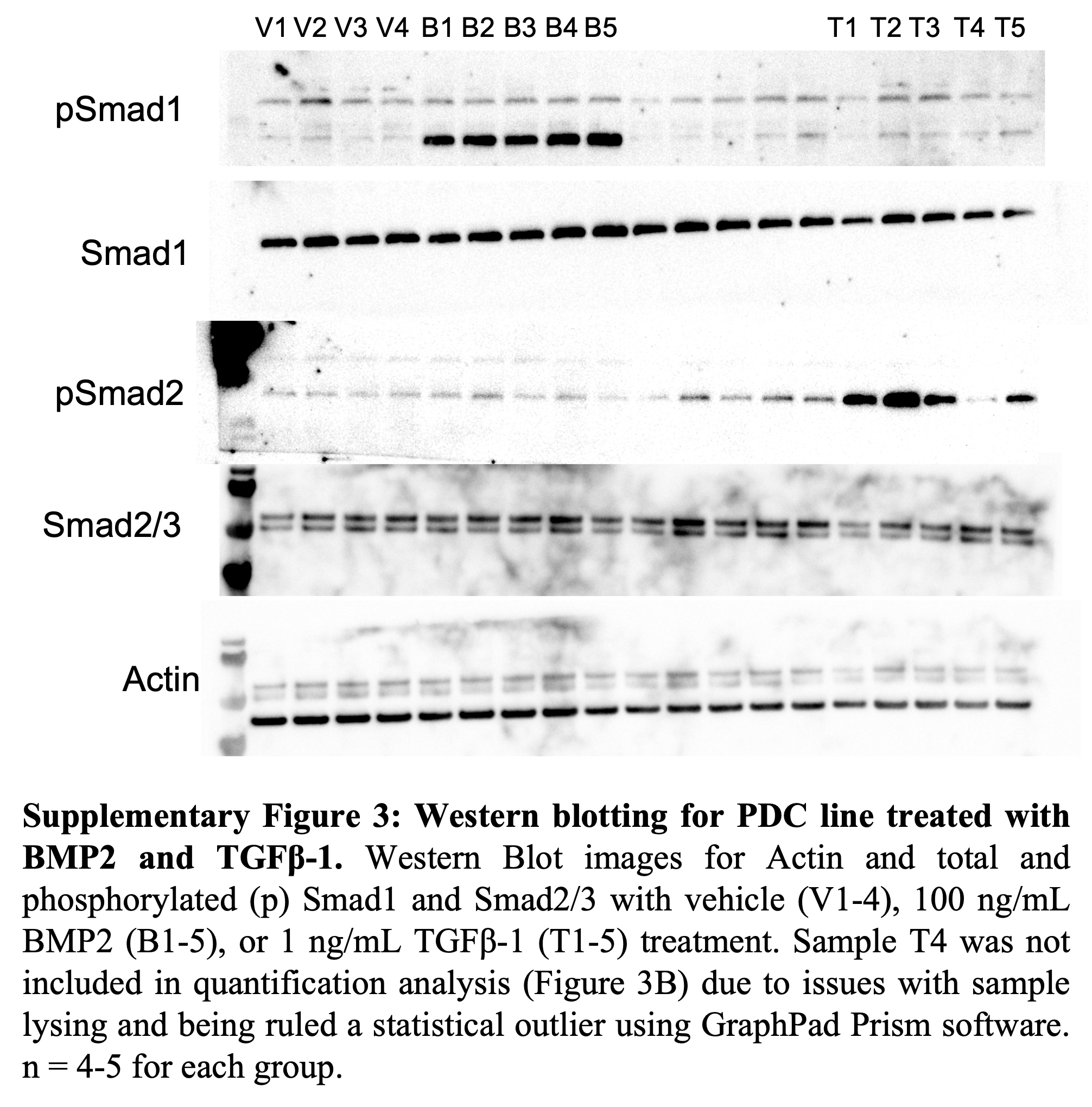

Supplement: Supplementary file 2 [file Image3.TIF]

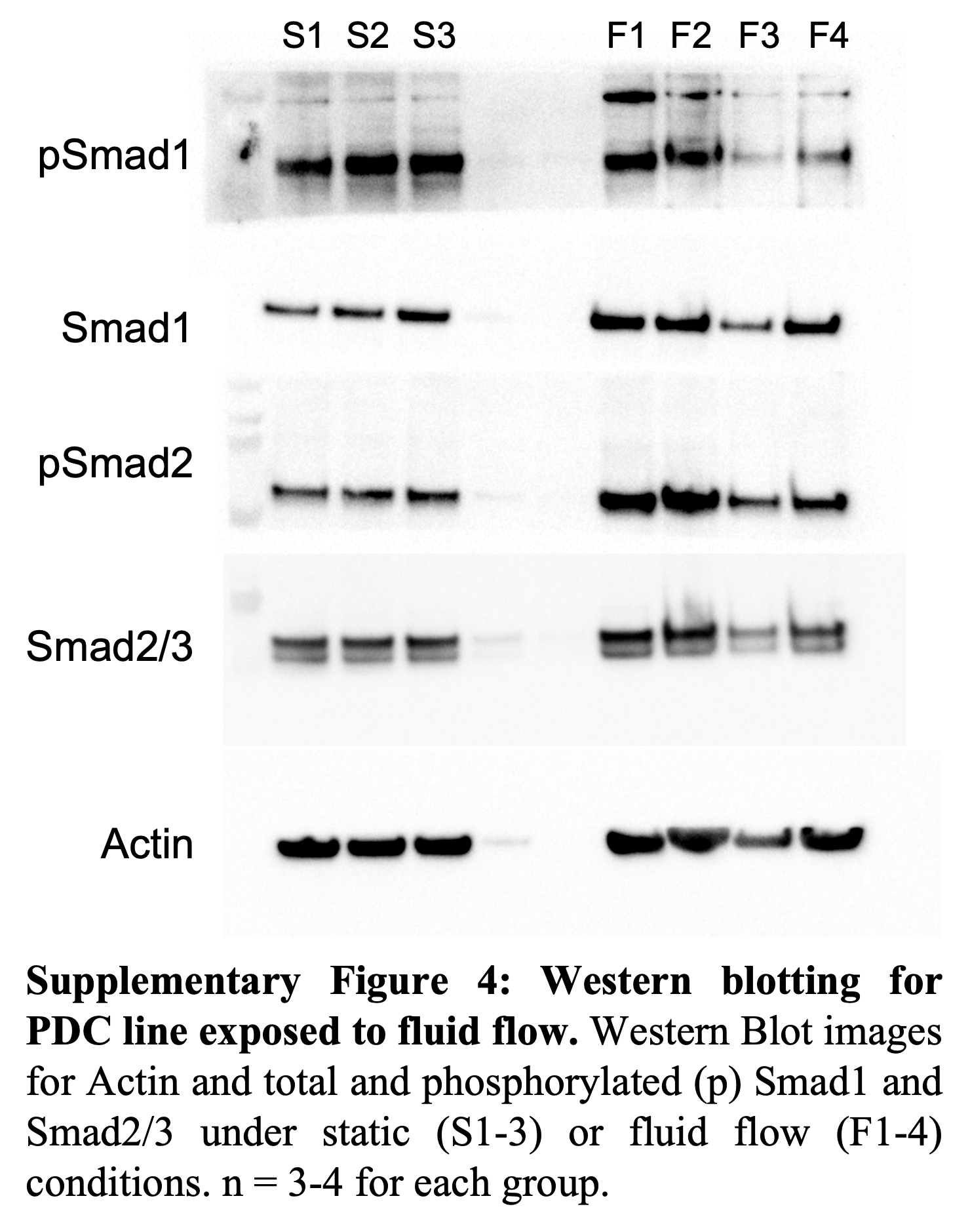

Supplement: Supplementary file 3 [file Image4.TIF]

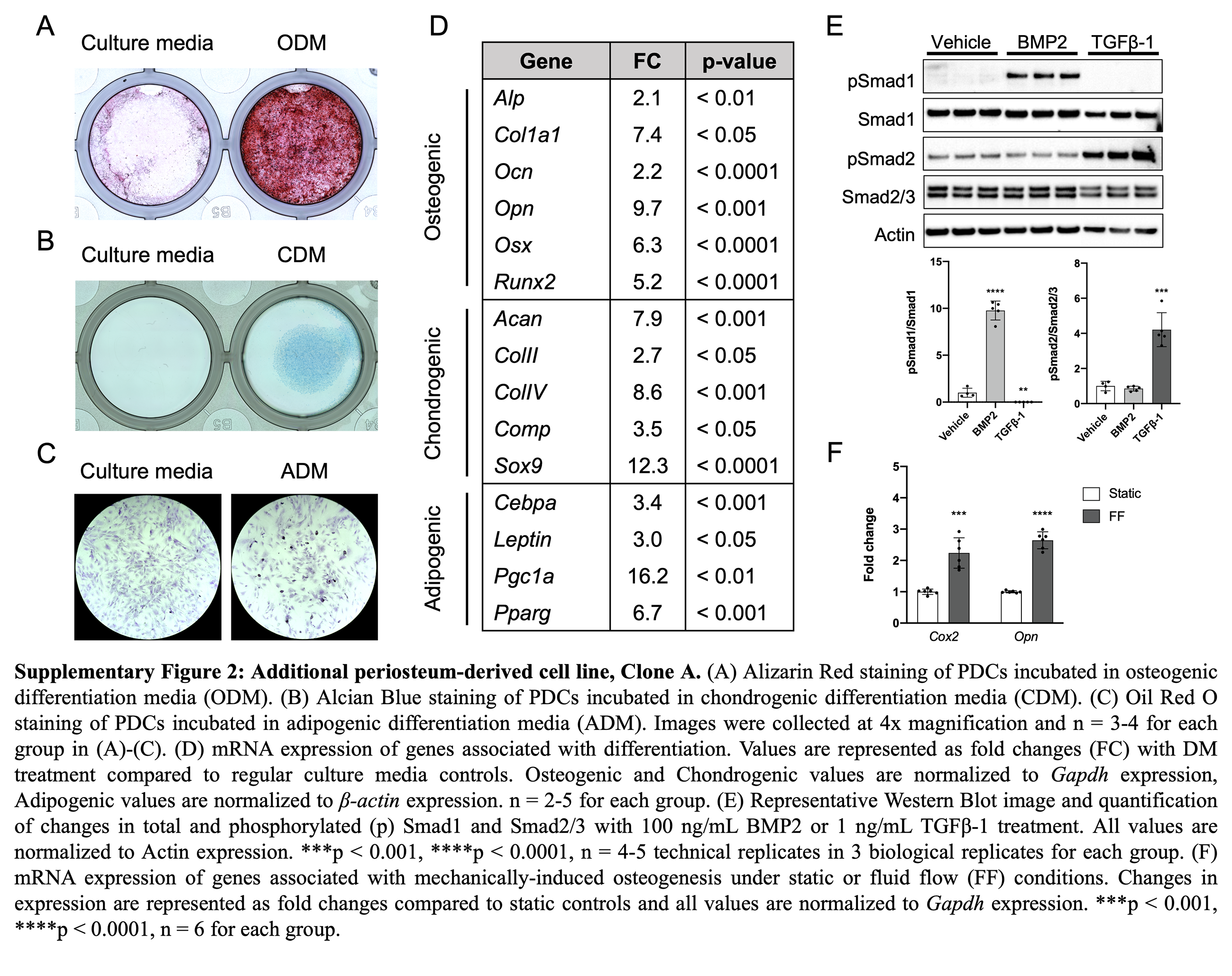

Supplement: Supplementary file 4 [file Image2.TIF]

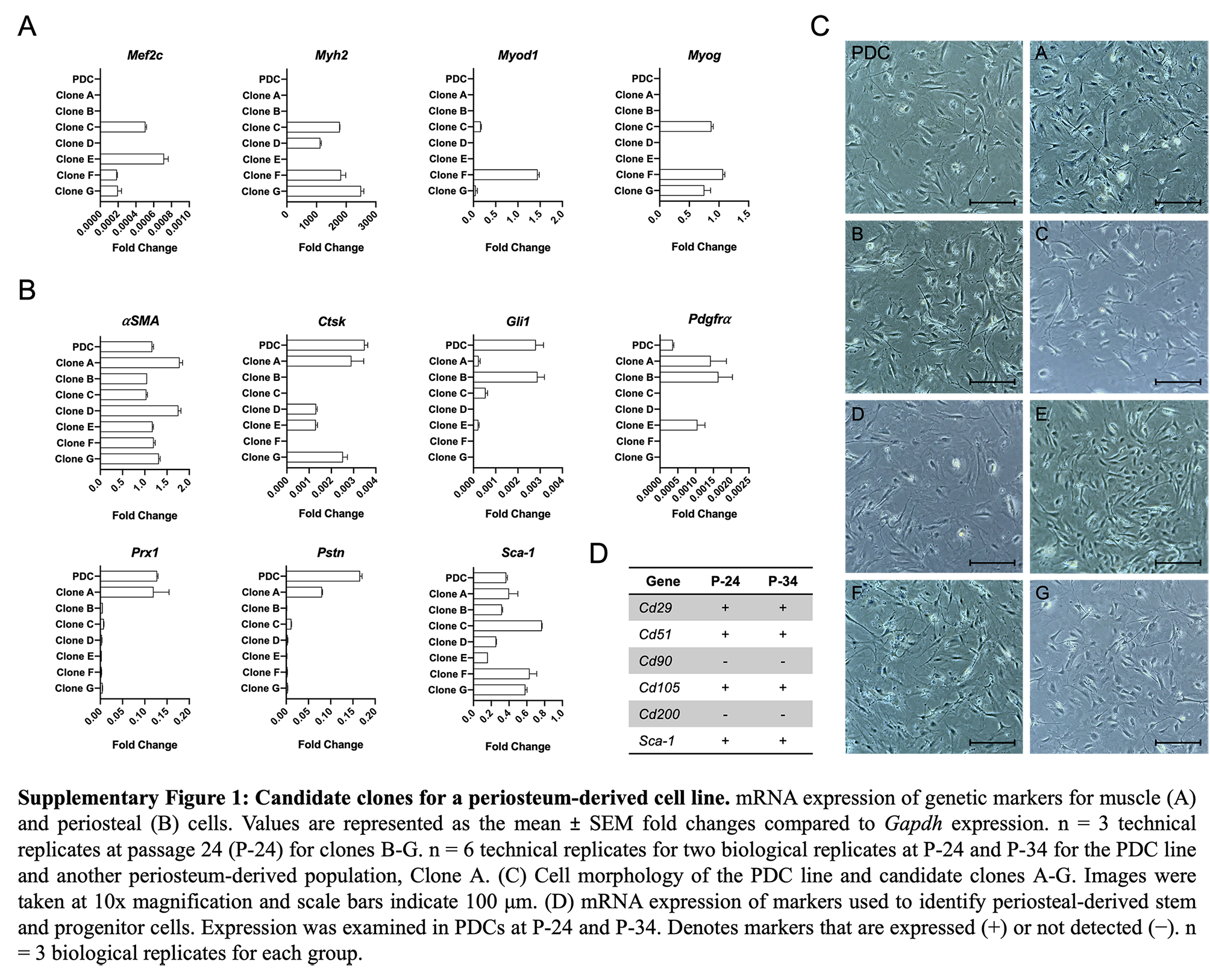

Supplement: Supplementary file 5 [file Image1.tif]

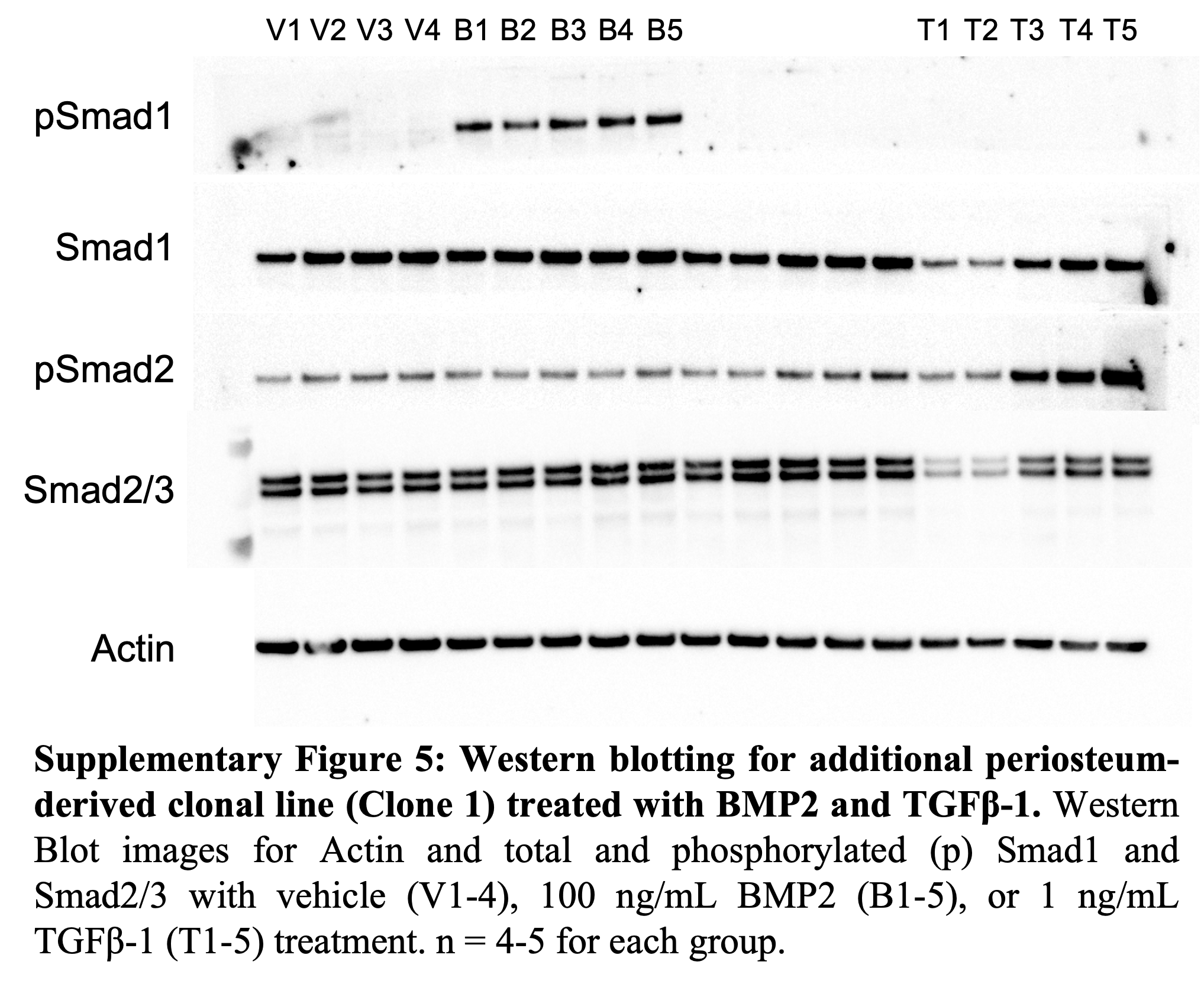

Supplement: Supplementary file 6 [file Image5.TIF]
